# Cytosolic localization of MBL-1/Muscleblind may be required for ectopic neurite outgrowth in a sensitized background in *C. elegans*

**DOI:** 10.17912/micropub.biology.002057

**Published:** 2026-05-07

**Authors:** Ho Ming Terence Lee, Chaogu Zheng

**Affiliations:** 1 School of Biological Sciences, The University of Hong Kong, Pokfulam, Hong Kong SAR, China

## Abstract

The evolutionarily conserved RNA-binding protein Muscleblind can function as both a splicing regulator in the nucleus and a mRNA stabilizer in the cytosol. *C. elegans* *mbl-1/* Muscleblind undergoes alternative splicing to generate long and short isoforms that contain one or two KR motifs needed for nuclear localization. We generate three alleles that express MBL-1 proteins with two, one, or no KR motifs and find that the proteins with two KR motifs are restricted in the nucleus and could not promote neurite growth in a sensitized background. Surprisingly, proteins with one or no KR motifs are located in both cytoplasm and nucleus.

**Figure 1. Cytoplasmic localization of MBL-1 may be required for its function in promoting neurite growth:** (A) Gene structure of *mbl-1* and the molecular change of various alleles. Transcription of *mbl-1* can start from exon 1 or exon 3. Exon 7 and 8 can be included or skipped to generate the long or short isoforms of *mbl-1* through alternative splicing. Expected splicing pattern of *mbl-1* gene from the three newly generated alleles. A forward primer binding to exon 3 and a reverse primer binding to exon 9 were used to amplify the *mbl-1* cDNA. The expected sizes of the PCR fragments were listed. The results of the RT-PCR using the *mbl-1* primers (F and R) and cDNA libraries prepared from different mutants are shown on the right. (B) Fluorescent images of TRNs (labelled by *uIs115[mec-17p::TagRFP]* ) in *mec-7(u278)* and *mec-7(u278); mbl-1(unk276)* mutants. Scale bars, 100 μm. The quantification below showed the ALM-PN length in various strains. Three asterisks indicate *p* < 0.001 in a Dunnett’s test comparing the strains with *mec-7(u278)* . Wild-type animals do not have a prominent ALM-PN. (C) Genomic DNA of *mbl-1* from *unk249* , *unk344* , and *unk276* were cloned, fused with GFP, and expressed under the *mec-17* promoter. The fluorescent signals of GFP fusion with various MBL-1 mutants. The diffusive TagRFP signal labels the entire cell body. Scale bars, 5 μm. (D) RT-PCR results using primers specific for *pqn-52c* , *pqn-72b* , *lgc-22a* , and *kin-4h* isoforms and cDNA libraries from different *mbl-1* mutants. The images on the right show the isoform-specific primer spanning particular exons. The primer and another primer that binds to an exon common to all isoforms were used to conduct the PCR experiment. *ama-1* served as an internal control.

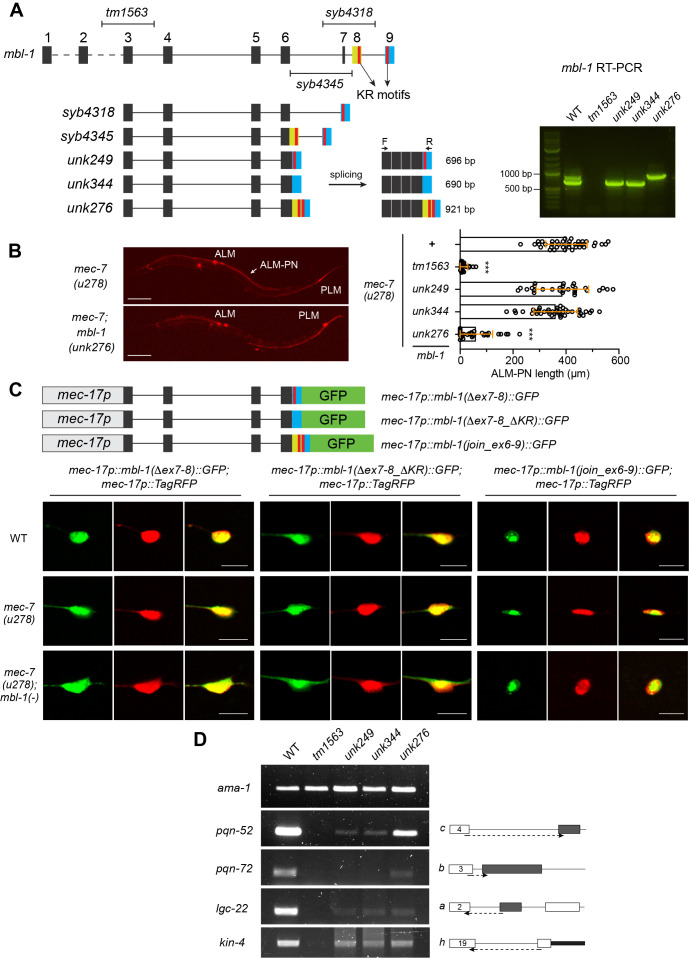



## Description

The Muscleblind family comprises a group of evolutionarily conserved RNA-binding proteins that play key roles in various aspects of RNA metabolism, most notably in the regulation of alternative splicing. A defining feature of these proteins is the presence of tandem zinc finger domains, each composed of three cysteine residues and one histidine residue (Fernandez-Costa et al., 2011). *Caenorhabditis elegans* has a single ortholog of Muscleblind protein, MBL-1 , which shows prominent expression in the nervous system. Loss-of-function studies have shown that *mbl-1* is essential for the synaptic formation at the neuromuscular junctions (Spilker et al., 2012), dendritic morphogenesis in PVD sensory neurons (Xie et al., 2023), microtubule stability and axonal growth in touch receptor neurons (TRNs), and alternative splicing of terminal selectors like *mec-3* (Lee et al., 2024). In addition to its classic role as a regulator of RNA splicing, recent studies have shown that MBL-1 also modulates mRNA stability through direct binding to target transcripts (Puri et al., 2023; Verbeeren et al., 2023). Since certain MBL-1 isoforms possess a pair of nuclear localization signals (NLS) while other isoforms do not, the protein may function both as a splicing regulator in the nucleus and an mRNA stabilizer in the cytoplasm. To disentangle the two functions, we engineered three alleles that produced MBL-1 proteins exclusively with or without the NLS and found that its presence in the cytoplasm is likely necessary to promote axonal growth in the TRNs.

Previous studies identified a bipartite nuclear localization signal (NLS) in mammalian Muscleblind MBNL proteins, consisting of two repeats of lysine-arginine residues (KR motifs) that regulate their subcellular localization (Kino et al., 2015). This bipartite KR motif is evolutionarily conserved in *C. elegans* MBL-1 , with one located near the end of exon 8 and the second at the beginning of exon 9 (Verbeeren et al., 2023). Since exon 8 can be selectively included in some but not all isoforms, the gene can code for MBL-1 isoforms with one or two KR motifs ( Fig. 1A ). To perturb the NLS in MBL-1 , Verbeeren et al. previously generated the *mbl-1 ( syb4318 )* and *mbl-1 ( syb4345 )* alleles. The *syb4318* allele featured a deletion of exon 7 and 8 and part of the flanking introns, resulting in the expression of isoforms with only one KR motif, whereas the *syb4345* allele involves a deletion of exon 7 and its flanking introns, leading to the expression of MBL-1 isoform with two KR motifs if the connected exons 6 and 8 are included in the mRNA. However, if the exon 6&8 is skipped, which would happen in the alternative splicing of *mbl-1* mRNA, the *syb4345* would still produces proteins with only one KR motif. Moreover, whether the single KR motif could still contribute to nuclear localization is unclear.

To address the above issues, we created three additional *mbl-1* alleles through CRISPR/Cas9-mediated gene editing. First, the *unk249* allele deleted exons 7 and 8 along with their flanking intronic sequences (thereby directly joining exons 6 and 9) and produced MBL-1 proteins with only one KR motif. Second, the *unk344* allele was built on top of *unk249* by further deleting the remaining KR motif, thus generating MBL-1 proteins with no KR motifs. Third, the *unk276* allele, in which exon 7, the introns flanking exon 7, and the intron between exons 8 and 9 were deleted, resulting in the fusion of exon 6, 8, and 9 and the production of MBL-1 proteins with two KR motifs only ( Fig. 1A ). We conducted RT-PCR to examine and sequence the transcripts of *mbl-1* in animals carrying the three alleles and confirmed that the gene editing indeed changed the sequence of the *mbl-1* transcripts as expected ( Fig. 1A ).

To understand the functional significance of these MBL-1 isoforms in promoting axonal growth, we crossed the above *mbl-1* alleles into the *mec-7 ( u278 )* mutants, which served as a sensitized background to test the effects on neurite growth. *mec-7* codes for a TRN-specific β-tubulin, and the *u278* (C303Y) is a gain-of-function mutation that led to the growth of a very long, ectopic posteriorly directed neurite in the ALM neurons (termed as ALM-PN) (Zheng et al., 2017). This ALM-PN does not exist or is very short in the wild-type animals. We previously found that the loss of *mbl-1* completely suppressed the growth of ALM-PN in the *mec-7 ( u278 )* mutants, suggesting that MBL-1 promotes neurite growth (Lee et al., 2024). Similar to the *mbl-1* null allele, the *unk276* allele (which only produces MBL-1 proteins with two KR motifs) also suppressed the ALM-PN growth ( Fig. 1B ), suggesting that the cytoplasmic presence of MBL-1 is likely required for its activity in promoting neurite growth. Both *unk249* and *unk344* alleles (which produces MBL-1 proteins with one or no KR motif) failed to suppress ALM-PN growth, suggesting that the KR motifs and nuclear localization may not be required for MBL-1 's function in inducing neurite growth in the *mec-7 ( u278 )* background.

To confirm the subcellular localization of the MBL-1 proteins produced by the above three alleles, we cloned the *mbl-1* gene from the mutants, fused them with GFP-coding sequences, and expressed the fusion proteins under the TRN-specific *mec-17* promoter. These reporters were introduced into the wild-type, *mec-7 ( u278 )* , and *mec-7 ( u278 ) mbl-1 (-)* animals. As expected, the proteins with two KR motifs were restricted to the nucleus, whereas the proteins with one KR motif showed diffusive expression throughout the TRN cell body ( Fig. 1C ). To our surprise, MBL-1 proteins with no KR motifs still showed a diffusive localization pattern in the cells and was not excluded from the nucleus. This result hinted that MBL-1 may be able to enter the nucleus in a mechanism that is independent of the two KR motifs. The shorter isoforms (which skipped exon 7 and 8) likely have both cytoplasmic and nuclear localizations.

To confirm that the nucleus-localized MBL-1 produced by the *unk276* allele is capable of splicing target genes, we analyzed four known MBL-1-regulated splicing events (Lee et al., 2024). The *unk276* animals could promote the normal splicing of *pqn-52c* , *pqn-72b* , *lgc-22a* , and *kin-4h* isoforms, suggesting that the long MBL-1 isoform is functional in controlling mRNA splicing. The *mbl-1 ( tm1563 )* deletion mutants served as a negative control ( Fig. 1D ). The MBL-1 proteins with one or no KR motifs produced by *unk249* and *unk344* alleles, respectively, could not promote the normal splicing of *pqn-52c* and *pqn-72b* but was able to promote the splicing of *lgc-22a* and *kin-4h* to the same extent as MBL-1 produced by *unk276* . Thus, the short MBL-1 isoform may still possess some ability to regular nuclear splicing, which is consistent with their nuclear localization.

MBL-1 is known to interact and stabilize the mRNAs of microtubule-related genes (such as *mec-17* /tubulin acetyltransferase, *mec-7* /β-tubulin, and *mec-12* /α-tubulin) (Puri et al., 2023), and we suspect that this mRNA-stabilizing role in the cytoplasm is essential for MBL-1 's function in promoting microtubule stability and neurite growth. However, since we were not able to generate a version of MBL-1 that is exclusively cytoplasmic, it remains unclear whether its nuclear localization is also required for its function in neurite extension. Our previous work found that MBL-1 promotes the splicing of *mec-3* , which activates the expression of *mec-17* , *mec-7* , and *mec-12* . It is possible that MBL-1 's canonical function as a splicing regulator in the nucleus also contribute to microtubule stabilization and neuronal morphogenesis.

## Methods

To generate the three *mbl-1* mutant alleles, we used CRISPR/Cas9-mediated genome editing to introduce double-strained breaks at two targeted sites (exons 6 and 9) in the endogenous *mbl-1* locus. Specifically, pairs of single guide RNAs (sgRNAs) were synthesized using the EnGen sgRNA Synthesis Kit (NEB, E3322V). A total of 1 μg of each sgRNA pair, combined with 20 pmol of recombinant Cas9 protein (EnGen S. pyogenes Cas9 NLS, NEB, M0646T), was microinjected into the gonads of young adult *C. elegans* . For precise editing of selected exons, we followed an established protocol that uses single-stranded DNA oligonucleotides (0.1 μg/μl) as homologous repair templates (Dokshin et al., 2018). To prevent re-cleavage by Cas9, synonymous mutations were incorporated into the repair templates at the protospacer-adjacent motif (PAM) sites.

To create TRN-specific fluorescent reporter constructs for the three *mbl-1* mutant alleles, we amplified the corresponding mutant genomic sequences and inserted them into a vector downstream of a 1.9 kb *mec-17* promoter and in-frame with GFP using Gibson Assembly (ClonExpress II One Step Cloning Kit, Vazyme Biotech, Nanjing, China). These plasmid constructs were then microinjected into the gonads of young adult *C. elegans* to generate transgenic lines carrying extrachromosomal arrays.

To conduct RT-PCR, total RNA was extracted from L4 animals using TRIzol reagent (Thermo Fisher). cDNA libraries were prepared through reverse transcription of the total RNA using SuperScript II Reverse Transcriptase with oligo(dT)s (Thermo Fisher). Four candidates were selected for semi-quantitative RT-PCR using isoform-specific primers based on previous studies (Lee et al., 2024). *ama-1* was used as an internal control for RT-PCR.

Fluorescence imaging was performed on a Leica DMi8 inverted microscope equipped with a Leica K5 monochrome camera. Images were acquired and analyzed using Leica Application Suite X software (version 3.7.2.22383). Measurements of ALM-PN length were obtained from day-1 adult animals cultivated at 20 °C, with at least 20 individuals scored per genotype.

## Reagents

**Table:** 

**Strain**	**Allele**	**Full Genotype**
CGZ1032	*uIs115*	*uIs115 [mec-17p::TagRFP] IV *
TU4879	*mec-7 ( u278 ) *	*mec-7 ( u278 ) X; uIs115 [mec-17p::TagRFP] IV *
TU6020	*mec-7 ( u278 ) mbl-1 ( tm1563 ) *	*mec-7 ( u278 ) X; mbl-1 ( tm1563 ) X; uIs115 [mec-17p::TagRFP] IV *
CGZ2408	*mec-7 ( u278 ) mbl-1 (unk249) *	*mec-7 ( u278 ) X; mbl-1 (unk249) X; uIs115 [mec-17p::TagRFP] IV *
CGZ2825	*mec-7 ( u278 ) mbl-1 (unk344) *	*mec-7 ( u278 ) X; mbl-1 (unk344) X; uIs115 [mec-17p::TagRFP] IV *
CGZ2654	*mec-7 ( u278 ) mbl-1 (unk276) *	*mec-7 ( u278 ) X; mbl-1 (unk276) X; uIs115 [mec-17p::TagRFP] IV *
CGZ2660	*unkEx898*	* unkEx898[mec-17p-mbl-1(del_ex7-8)-GFP-unc-54-3'utr; ceh-22p::GFP]; uIs115 [mec-17p::TagRFP] IV *
CGZ2720	*mec-7 ( u278 ); unkEx898 *	*mec-7 ( u278 ) X; unkEx898[mec-17p-mbl-1(del_ex7-8)-GFP-unc-54-3'utr; ceh-22p::GFP]; uIs115 [mec-17p::TagRFP] IV *
CGZ2715	*mec-7 ( u278 ) mbl-1 ( tm1563 ); unkEx898 *	*mec-7 ( u278 ) X; mbl-1 ( tm1563 ) X; unkEx898[mec-17p-mbl-1(del_ex7-8)-GFP-unc-54-3'utr; ceh-22p::GFP]; uIs115 [mec-17p::TagRFP] IV *
CGZ2716	*unkEx899*	* unkEx899[mec-17p-mbl-1(join_ex6-9)-GFP-unc-54-3'utr; ceh-22p::GFP]; uIs115 [mec-17p::TagRFP] IV *
CGZ2717	*mec-7 ( u278 ); unkEx899 *	*mec-7 ( u278 ) X; unkEx899[mec-17p-mbl-1(join_ex6-9)-GFP-unc-54-3'utr; ceh-22p::GFP]; uIs115 [mec-17p::TagRFP] IV *
CGZ2661	*mec-7 ( u278 ) mbl-1 ( tm1563 ); unkEx899 *	*mec-7 ( u278 ) X; mbl-1 ( tm1563 ) X; unkEx899[mec-17p-mbl-1(join_ex6-9)-GFP-unc-54-3'utr; ceh-22p::GFP]; uIs115 [mec-17p::TagRFP] IV *
CGZ2946	*unkEx1041*	* unkEx1041(mec-17p-mbl-1(del_ex7-8_KR-motifs)-GFP-unc-54-3'utr; ceh-22p::GFP); uIs115 [mec-17p::TagRFP] IV *
CGZ2912	*mec-7 ( u278 ); unkEx1041 *	*mec-7 ( u278 ) X; unkEx1041(mec-17p-mbl-1(del_ex7-8_KR-motifs)-GFP-unc-54-3'utr; ceh-22p::GFP); uIs115 [mec-17p::TagRFP] IV *
CGZ2947	*mec-7 ( u278 ) mbl-1 ( tm1563 ); unkEx1041 *	*mec-7 ( u278 ) X; mbl-1 ( tm1563 ) X; unkEx1041(mec-17p-mbl-1(del_ex7-8_KR-motifs)-GFP-unc-54-3'utr; ceh-22p::GFP); uIs115 [mec-17p::TagRFP] IV *

**Table:** 

**CRISPR Reagents**	**Sequence**	**Purpose**
mbl-1-crispr-ex6	TCAAGACCCTTATACAGCAG	Exon 6 target site - wild type background
mbl-1-crispr-ex9	GCTCCGTTCTTGTCGAGAGT	Exon 9 target site - wild type background
mbl-1-repair-del8	TACTACAACGGCATGATGTATCCACAAGTA CTACAGGATCCATACACTGCTGCGGCAGTGA ATCAG GGAGCTGTACCAATGAAGCGACCAA CACTGGATAAAAATGGTGCAATGTTATACTC ACCGGTAGCTCAGCAGGC	Repair templates - joining of exon 6 and 9 together (removal of exon 8 and adjacent introns)
mbl-1-crispr-ex6_2	TATGGATCCTGTAGTACTTG	Exon 6 target site - unk249 background
mbl-1-crispr-ex9_2	ACCAATGAAGCGACCAACAC	Exon 9 target site - unk249 background
mbl-1-repair-ex8	TACCCACCCTACTACAACGGCATGATGTATC CACAAGTACTTCAAGACCCTTATACAGCAGC GGCAGTGAATCAGCAGCTACAAACTGCCGC CTTGCTTGGCAACGTCGGAGGACTGCTTTC GGCTC AATCGGCGGCCGCCTTCATGGCCAA CTCGTCGGCAGCGGCTGCAGCAGCCCAACA AACGCCCT CACCGTTGCTTCGTCTGCAAAG GAAACGAGCGCTGGAAGAGGAGAACACGA ATGGCAACGATATGACGTCAGCAGCAGCGG CTCACACACAATTGCTCTCATTGGCCGCGG GAGCTGTACCAATGAAGCGACCAACTCTCG ACAAGA ACGGAGCAATGTTATACTCACCGG T	Repair templates - insert exon 8 between exon 6 and 9
mbl-1-repair-ex9	TACCCACCCTACTACAACGGCATGATGTATC CACAAGTACTTCAAGACCCTTATACAGCAG CGGCAGTGAATCAGGGAGCTGTACCAATGC CAACTCTCGACAAGAACGGAGCAATGTTAT ACTCACCGGTAGCTCAGCAGGCACAACAATT	Repair templates - removal of KR motifs on exon 9

**Table:** 

**Primers**	**Sequence**	**Purpose**
S-mbl-1-ex6-F	ccgttccagCAACAACAAGC	CRISPR sequencing
S-mbl-1-3'UTR-R	attcacatgactagcctcccag	CRISPR sequencing
m17p-mbl-1-F	tgtgagacgattcgatcATGTTCGACGAAAACAGTAATGCCG	TRN-specific fluorescent reporter constructs cloning
mbl-1-GFP-R	TTCTCCTTTACTGAATGGTGGTGGCTGCATGT	TRN-specific fluorescent reporter constructs cloning
mbl-1_ATG_F	ATGTTCGACGAAAACAGTAATGCCG	*mbl-1* RT-PCR
mbl-1_TAG_R	CTAGAATGGTGGTGGCTGCATG	*mbl-1* RT-PCR
ama-1-cDNA-F	CGGAGGAGATTAAACGCATGTC	*ama-1* RT-PCR
ama-1-cDNA-R	CGAGCTCCGTTTTCTCTAATAATATACTTG	*ama-1* RT-PCR
kin-4-cDNA-f	AACTTGTTACGTGATGTACCCTTCTG	*kin-4h* RT-PCR
kin-4h-cDNA-r	TGGCGATGGACTTCTCTATCTCATTT	*kin-4h* RT-PCR
pqn-52-cDNA-f	CTCCATCGGACATCCGAATTCC	*pqn-52c* RT-PCR
pqn-52-cDNA-r	GTGGTTTTTCTTGGGACTGTCC	*pqn-52c* RT-PCR
pqn-72-cDNA-f	TCGGAACTTTATACGTCAGCAGTT	*pqn-72b* RT-PCR
S-pqn-72-r	TTTCGATGGAACTCGATGAGTC	*pqn-72b* RT-PCR
lgc-22-cDNA-f	CGTTGAAGTTGTGTCAATTACCCACT	*lgc-22a* RT-PCR
S-lgc-22-r	ACAGTGGATAAAGCGAAGATGACG	*lgc-22a* RT-PCR
